# Procreative sex in infertile couples: the decay of pleasure?

**DOI:** 10.1186/1477-7525-10-140

**Published:** 2012-11-23

**Authors:** Roberto Marci, Angela Graziano, Isabella Piva, Giuseppe Lo Monte, Ilaria Soave, Emilio Giugliano, Silvia Mazzoni, Roberta Capucci, Maria Carbonara, Stefano Caracciolo, Alfredo Patella

**Affiliations:** 1 Department of Biomedical Sciences and Advanced Therapies, Section of Obstetrics and Gynecology, University of Ferrara, Corso Giovecca 183, 44121, Ferrara, Italy; 2 Department of Experimental and Clinical Medicine, Section of General and Clinical Psychology, University of Ferrara, Ferrara, Italy

**Keywords:** Infertility, Psychology, Sexuality, Sexual dysfunction, Assisted Reproductive Technology, Sexual disorders, Sexual behavior, Psycho-sexology

## Abstract

**Background:**

Infertility represents a major challenge to the emotional balance and sexual life of couples, with long-lasting and gender-specific effects. The objective of this study is to explore personality features of infertile patients and detect possible sexual disorders in couples undergoing infertility treatment.

**Materials and methods:**

In this prospective study 60 infertile couples and 52 fertile control couples were asked to complete standardized and validated questionnaires: the Adjective Check List (ACL) to enquire about personality features and the Female Sexual Function Index (FSFI) or the International Index of Erectile Function (IIEF) to assess sexual functioning of female and male partners. The study population was divided into 3 groups: Group A (N = 30, recently diagnosed infertile couples) Group B (N = 30, infertile couples already undergoing Intrauterine Insemination) and Group C (N = 52, fertile control group).

**Results:**

Infertile patients did not display any distinguishing personality features. Regarding sexual function, men of all the three groups scored higher in both questionnaires (sexual satisfaction, desire and orgasm) than their female partners. Comparing results between groups, Group A male partners obtained lower scores in all the subscales. Women belonging to Group A and Group B showed an impairment of sexual arousal, satisfaction, lubrification and orgasm when compared to fertile controls.

**Conclusions:**

Even if at the very first stages of infertility treatment no personality disturbances can be detected, the couples’ sexual life is already impaired with different sexual disorders according to gender.

## Background

Infertility is defined as the inability to conceive after 12 months of unprotected sexual intercourse [1]. The link between infertility and sexuality is complex and bi-directional: infertility can be considered either as a cause or a consequence of a sexual dysfunction. However, organic sexual dysfunctions are regarded as a minor cause of infertility, with a conservatively estimated rate of about 5% of all infertility cases [2]. Furthermore, male sexual disorders such as chronic erectile dysfunctions and failure to ejaculate make conception impossible, whereas only vaginismus as a female sexual disorder prevents pregnancy [3]. On the other hand, according to a recent review, sexual disorders – depending on both infertility itself and infertility diagnosis and treatment – are common in infertile couples, with women more affected than men [4]. As far as infertility is concerned, sexual intercourse can lose its spontaneity because it is aimed at “baby-making” and it is strictly restricted to “fertile” days. Sexuality can be therefore deprived of its recreative and erotic value and subordinated to the direct goal of pregnancy [5]. This distortion of a sexual relationship can be long lasting and even cause disruptions to sexual life, once involuntary childlessness proves to be permanent [6]. Furthermore, sexual intercourse may be increasingly associated with a sense of failure, affecting even the patient’s image of his or her own body. As far as Medical Assisted Procreation (PMA) is concerned, it can affect sexuality in several ways: medical vocabulary can be traumatizing and medical procedures can arouse a sense of anxiety and consequent temporary erectile dysfunctions. Medical vocabulary can be hurtful and it can contribute to the impairment of male sexual identity [7,8]. Medical prescriptions in case of hormonal stimulation can enhance the tendency to have sex on fertile days [5,6]. The resulting “sex by the clock” can be stressful, especially for male partners, who feel progressively degraded to the role of “sperm donors”. In women, hormone medication itself as well as certain side effects of hormonal therapy (mood swings and weight gain) can alter their sexual behavior and their experience of sexuality [4].

Likewise, infertility could either cause or derive from psychosocial diseases. The dualism between the so-called “Psychogenic hypothesis” and the hypothesis of “Psychological consequences of infertility” has been solved with the proposal of an interactive and bidirectional relationship between infertility and psychosocial diseases [9,10]. Even if the psychogenic hypothesis is now rejected by most researchers, the psychodynamic perspective has been partially replaced by stress and coping models [9,11]. The ability to cope with stress or potentially stressing stimuli depends on the individual’s personality and defense mechanisms [12]. Some investigators have found that infertile couples have an unusual personality profile, which might be a cause as well as a consequence of infertility [13]. Tendency to reserve, introversion, mistrust, anxiety, guilt feeling, excessive attachment to one’s own ideas, poor availability and flexibility, and excessive concern about formality were significantly more marked in infertile partners than in fertile controls [14,15]. However only few studies have assessed the psychosocial and personality features related to infertility, with contradictory results [13].

The aim of this prospective study is to assess the consequences of infertility diagnosis and treatment on sexual behavior. Infertile couples were compared to control couples in order to detect to what extent the couples sexuality was affected by infertility. Furthermore, since this study involved both partners, we focused our attention on any possible differences of gender on sexual life. Finally, through the analysis of a questionnaire on personality characteristics, we tried to find out possible links between sexual disturbances and personality dimensions as well as concurrent levels of distress in the patients.

## Materials and methods

### Samples

The study was carried out by the Infertility Unit of Ferrara University between October 2011 and May 2012. We included couples aged 18 to 45, with a good knowledge of the Italian language. We excluded couples previously diagnosed with psychiatric pathologies according to DSM-IV classification (Diagnostic and statistical manual of mental health disorders, 4th edition) [16] as well as patients suffering from any organic disease possibly responsible for altered sexual behavior.

The study population consisted of 112 couples and all participants completed a set of questionnaires. All participants signed an informed consent and ethical approval was obtained from the Local Ethics Committee. Male and female partners of each couple filled in their questionnaires separately in the presence of a physician.

The population was divided into 3 groups:

Group A: 30 recently (within two months) diagnosed infertile couples that completed the questionnaires at their first clinic appointment. We only included couples affected by primary infertility (i.e. they had not had any children before, either with the current partner or with others). On the contrary, couples suffering from secondary infertility (i.e. with previous children) were excluded because this might be a protective factor against emotional distress, as observed in other studies [17].

Group B: 30 infertile couples already undergoing IUI at the time of the administration of questionnaires. These couples were selected on the grounds of the same criteria as Group A.

Group C: 52 co-habitant couples in fertile age. Women presenting for routine checks and smear test screening at the Gynecologic General Service were asked to join the study together with their partners. The couples did not have any known infertility problems. The couples were not intentionally looking for pregnancy at the time of their enrollment (i.e. they were not having sex on fertile days on purpose), but they did not exclude the possibility of a pregnancy then or in the future. In fact we could neither ascertain nor rule out the use of contraceptives in the control group.

### Measures

All patients from the 3 groups completed a set of self-reported indices: the Adjective Check List (ACL) questionnaire was used to evaluate the personality characteristics of the patients and the International Index of Erectile Function (IIEF) or the Female Sexual Function Index (FSFI) to assess the sexual functionality of both the male and female partner.

ACL is made up of 37 subscales exploring five specific areas: modus operandi, needs, originality and intelligence, distinguishing features and lastly some information for the patients’ transactional analysis [18-22]. The Adjective Check List (ACL), as a personality measure, identifies common psychological traits, but it doesn't measure psychopathological disorders. The 20 subscales we have considered were selected on the grounds of their relevance to the aim of the study, in order to assess the personality traits that influence sexual activity [23] (Table 1). The subscales dealing with personality traits that were more likely to be affected by infertility were chosen. Both partners are asked to choose the adjective that best describes their personality out of a list of 300 adjectives defining different behavioral styles.

**Table 1:** Mean comparisons using Kruskal Wallis test on ACL

**ACL subscales**	**Group A (n = 30)**		**Group B (n = 30)**		**Group C (n = 52)**		***P***
	**M**	**SD**	**M**	**SD**	**M**	**SD**	
Ach	51.20	12.76	48.23	7.61	50.96	13.50	NS
Dom	49.43	13.43	49.70	8.45	50.90	12.36	NS
End	49.50	10.23	49.27	9.37	51.27	10.23	NS
Ord	48.43	10.61	48.40	12.12	49.63	10.01	NS
Int	53.60	7.75	49.77	8.15	53.04	10.04	NS
Nur	51.40	9.44	48.47	9.93	48.54	11.04	NS
Aff	50.47	10.42	49.10	11.39	49.75	11.13	NS
Het	50.90	8.20	50.90	10.61	50.13	9.80	NS
Exh	46.83	10.01	49.27	9.18	50.23	9.97	NS
Aut	46.00	8.83	47.40	8.87	48.29	11.61	NS
Agg	51.23	10.54	51.80	10.57	52.90	10.17	NS
Cha	48.50	7.39	45.90	7.53	48.98	9.01	NS
Suc	47.57	10.67	47.53	9.05	46.58	9.56	NS
Aba	48.63	10.93	48.23	8.57	46.29	9.54	NS
Def	49.57	9.81	49.77	9.10	48.79	10.65	NS
S-Cfd	48.10	12.31	48.33	8.51	51.35	11.61	NS
P-Adj	53.27	9.86	50.47	10.69	51.13	11.21	NS
Iss	49.27	12.04	49.57	10.26	52.00	7.48	NS
Mas	48.00	11.19	48.78	6.37	47.73	10.54	NS
Fem	51.22	8.66	49.07	12.66	48.04	9.04	NS

M: mean value; SD: standard deviation; Ach: need for success; Dom: need for dominance; End: need for endurance; Ord: need for order; Int: need to understand the others; Nur: nursing need; Aff: affiliation need; Het: need to establish heterosexual relationships; Exh: exhibition need; Aut: need for autonomy; Agg: need to be aggressive; Cha: need for change; Suc: succour need; Aba: abatement need; Def: need to show deference; S-Cfd: self confidence; P-Adj: Personal Adjustment; Iss: high self-esteem; Mas: male orientation; Fem: female orientation.

IIEF is a 15-item measure of different aspects of male sexual functioning (erectile function, orgasm, sexual desire, sexual satisfaction and overall satisfaction) [24,25]. The questionnaire is meant to detect the possible presence and severity of sexual dysfunction, while the other subscales provide only qualitative information and do not have a cut-off value. The original English instrument has been validated in twelve countries and translated in ten languages, including Italian. The severity of sexual dysfunction was evaluated on a 0–30 point score system. The score must be interpreted as follows:

0–10: severe sexual dysfunction

11–16: moderate sexual dysfunction

17–25: mild sexual dysfunction

26–30: no sexual dysfunction.

FSFI is a multidimensional self-report instrument to assess female sexual functionality. This measure has been validated on a sample of women with a clinical diagnosis of sexual excitement disorder [26,27]. FSFI is made up of 19 items arranged in six subscales: sexual interest/desire, sexual arousal, lubrification, orgasm, sexual satisfaction and sexual pain. Every item is assigned a score that gives a final figure ranging from a minimum value of 2.0 to a maximum of 36.0.

### Statistical elaboration

Data were analyzed using SPSS, version 20.0 (SPSS Inc., Chicago, IL, USA). Non-parametrical analyses were applied because of the small sample size. Kruskal-Wallis test was used to assess the personality characteristics and sexual functionality of both the male and female partner among the three groups. Mann–Whitney U test was used to assess specific sexual functioning between the male and female partner in each group (in order to evaluate a possible gender difference). Differences in values of erectile dysfunctions were studied using Fisher exact test. Significance level was set at *P* < 0,05.

## Results

The women’s age range was between 28 and 40 years with a mean age of 34.64 ± 3.89 SD. No significant difference was observed between the three groups (group A: 34.60 ± 4.28 SD; group B: 35.33 ± 3.82 SD; group C: 34.26 ± 3.79 SD). The men’s age meanwhile ranged from 28 to 45 years with a mean of 38.12 ± 3.24 SD. No significant difference was also observed between the three groups (group A: 38.53 ± 2.87 SD; group B: 38.40 ± 3.35 SD; group C: 37.30 ± 3.45 SD). The mean duration of the partnership was not statistically different among the three groups.

We initially performed a non parametric analysis of variance in the 20 subscales of ACL among the three groups (Table 1). There was no significant difference between the three groups. This implied that the infertile patients did not show any distinguishing personality features. The sample could be therefore considered homogeneous with regards to the emotional profile and hence possible sexual disorders could not be due to any psychological problem but must be instead ascribed to infertility treatment.

Furthermore, Mann–Whitney U test was applied to compare the subscales “sexual desire”, “sexual satisfaction” and “orgasm” (included in both the FSFI and IIEF questionnaires), in order to point out a possible gender difference. A significant difference was subsequently detected with all the male patients scoring higher than their female partners independently of the groups they were taken from (Table 2).

**Table 2:** Comparison between the subscales “sexual desire”, “sexual satisfaction”, and “orgasm” in IIEF and FSFI questionnaires using Mann–Whitney U test

		**Male (n = 112)**		**Female (n = 112)**		***P***
		**M**	**SD**	**M**	**SD**	
Desire	Group A	7.47	1.38	4.48	0.88	<0.0005
	Group B	8.07	1.14	4.40	0.80	<0.0005
	Group C	8.85	1.07	4.75	0.88	<0.0005
Sexual Satisfaction	Group A	11.33	3.59	4.91	1.25	<0.0005
	Group B	11.60	1.81	5.36	0.55	<0.0005
	Group C	13.27	1.93	5.65	0.74	<0.0005
Orgasm	Group A	8.60	2.72	5.07	0.80	<0.0005
	Group B	9.20	1.62	5.12	0.75	<0.0005
	Group C	9.88	0.32	5.55	0.72	<0.0005

M: mean value; SD: standard deviation.

Finally, we evaluated possible differences in sexual function among the three groups. We perform on both FSFI and IIEF questionnaires, a non parametric analysis of variance using Kruskal Wallis test, and a multiple comparison using Mann–Whitney U test (Table 3 and Table 4). This approach enabled us to point out possible differences in sexual behavior depending on the stage of treatment.

**Table 3:** Mean comparisons using Kruskal Wallis Test on FSFI and IIEF

		**Group A (n = 30)**		**Group B (n = 30)**		**Group C (n = 52)**		***P***
		**M**	**SD**	**M**	**SD**	**M**	**SD**	
IIEF	Erectile	25.47	6.79	28.93	2.27	29.62	0.69	<0.0005
	Orgasm	8.60	2.72	9.20	1.62	9.88	0.32	0.03
	Desire	7.47	1.38	8.07	1.14	8.85	1.07	<0.0005
	Sexual Satisfaction	11.33	3.59	11.60	1.81	13.27	1.93	<0.0005
	Overall satisfaction	8.27	2.05	8.40	1.22	9.12	1.35	0.006
FSFI	Orgasm	5.07	0.80	5.12	0.74	5.55	0.72	0.002
	Desire	4.48	0.88	4.40	0.79	4.75	0.87	NS
	Arousal	4.92	0.93	4.96	0.64	5.62	0.41	<0.0005
	Lubrification	5.48	0.70	5.70	0.41	5.85	0.37	0.01
	Pain	5.28	1.43	5.20	1.11	5.61	0.65	NS
	Sexual Satisfaction	4.91	1.25	5.36	0.55	5.65	0.73	<0.0005

M: mean value; SD: standard deviation.

**Table 4:** Multiple comparisons among the three groups using Mann–Whitney U test on FSFI and IIEF

		**Group A (n = 30)**		**Group C (n = 52)**		***P***	**Group B (n = 30)**		**Group C (n = 52)**		***P***	**Group A (n = 30)**		**Group B (n = 30)**		***P***
		**M**	**SD**	**M**	**SD**		**M**	**SD**	**M**	**SD**		**M**	**SD**	**M**	**SD**	
IIEF	Erectile	25.47	6.79	29.62	0.69	<0.0005	28.93	2.27	29.62	0.69	NS	25.47	6.79	28.93	2.27	0.001
	Orgasm	8.60	2.72	9.88	0.32	0.007	9.20	1.62	9.88	0.32	NS	8.60	2.72	9.20	1.62	NS
	Desire	7.47	1.38	8.85	1.07	<0.0005	8.07	1.14	8.85	1.07	0.006	7.47	1.38	8.07	1.14	NS
	Sexual Satisfaction	11.33	3.59	13.27	1.93	0.002	11.60	1.81	13.27	1.93	<0.0005	11.33	3.59	11.60	1.81	NS
	Overall satisfaction	8.27	2.05	9.12	1.35	0.01	8.40	1.22	9.12	1.35	0.004	8.27	2.05	8.40	1.22	NS
FSFI	Orgasm	5.07	0.80	5.55	0.72	0.005	5.12	0.74	5.55	0.72	0.002	5.07	0.80	5.12	0.74	NS
	Desire	4.48	0.88	4.75	0.87	NS	4.40	0.79	4.75	0.87	NS	4.48	0.88	4.40	0.79	NS
	Arousal	4.92	0.93	5.62	0.41	0.001	4.96	0.64	5.62	0.41	<0.0005	4.92	0.93	4.96	0.64	NS
	Lubrification	5.48	0.70	5.85	0.37	0.009	5.70	0.41	5.85	0.37	0.01	5.48	0.70	5.70	0.41	NS
	Pain	5.28	1.43	5.65	0.73	NS	5.20	1.11	5.65	0.73	0.03	5.28	0.70	5.20	1.11	NS
	Sexual Satisfaction	4.91	1.25	5.65	0.73	<0.0005	5.36	0.55	5.65	0.73	0.003	4.91	1.25	5.36	0.55	NS

M: mean value; SD: standard deviation.

Kruskall Wallis test showed significant lower values, regarding IIEF questionnaire, in Group A for all of the subscales studied. Regarding the FSFI questionnaire, the subscales “orgasm”, “arousal”, “lubrification” and “sexual satisfaction” showed a significant difference among the three groups. Considering all of these parameters women of Group A and Group B displayed lower than average scores compare to controls (Table 3).

The Mann–Whitney U test applied to the IIEF questionnaire detected a significant difference in all the subscales between Group A and Group C. Instead, the comparison between Group B and controls showed significant differences in the parameters “desire”, “sexual satisfaction” and “overall satisfaction”. Finally, comparing Group A and Group B, only the subscale “erectile dysfunction” was significantly higher in Group B (Table 4).

The real erectile dysfunction, defined for values <26 at IIEF score, was found to be 26.6% in Group A, 6.66% in Group B and 0% in Group C (*P* <0.0005 using Fisher exact test).

Regarding FSFI, the Mann–Whitney U test showed values significantly lower in “orgasm”, “arousal”, “lubrification”, and “sexual satisfaction” subscales in Group A when compared to controls. Multiple comparisons between Group B and controls showed significant differences in all the subscales except for the parameter “desire”. There were not significant differences between Group A and Group B (Table 4).

## Discussion

The present study has provided useful data about the personality features and the sexual behavior of infertile couples.

Regarding the psychological dimension we did not find any distinctive feature in infertile patients. This finding is in accordance with the extensive review of Greil et al. that states that most studies have failed to uncover many personality differences between infertile and fertile groups [9]. However, in a recent study exploring individual psychological functioning and marital adjustment, infertile couples showed higher scores in measures of depression, external and internal shame, acceptance and self-compassion compared to fertile controls and adoption candidates. Furthermore, infertile patients pursuing medical treatment presented higher avoidant and emotional coping styles [28]. A possible reason for these contradictory results may be the lack of an adequate follow-up in all groups.

Our study did not point out any gender difference in the emotional profile of infertile patients, whereas previous studies have demonstrated that infertility is a more devastating experience for the female partner [29,30]. A Canadian survey, although dated, is still significant in this field, since it enrolled 449 couples. Women displayed higher distress than their partners on a global measure of psychiatric symptoms and in the subscales of anxiety, depression, hostility and cognitive disturbances as well as on measures of stress and self-esteem [31]. Besides, a two-year longitudinal study aimed at assessing the psychosocial impact of infertility confirmed that women experience substantially higher levels of stress than men at the time of diagnosis. However this gender difference is reduced in time [32]. Furthermore, a gender comparison between men and women candidate to in-vitro fertilization (IVF) showed a greater distress in the female partner. The negative emotional response was related both to infertility diagnosis and to infertility treatment. In addition to that women were more likely to endorse negative reactions to IVF failure [33]. It has been speculated that three factors operate together in driving the women distress level higher than their spouses [31]. First of all the social responsibility of conceivement and pregnancy is still attributed mainly to the female partner [32]. Furthermore medical treatment is more intrusive on women than men (time-counsuming, painful [31,32]. Finally coping strategies differ between men and women: men tend to deny and remain active, while women cannot imagine life with no children and develop depressive reactions [31,33].

As far as sexual functioning is concerned, we found significant differences both between men and women and among the groups. With regard to gender difference, women displayed lower scores in the subscales “orgasm”, “sexual satisfaction” and “desire”. This finding is in accordance with the results of previous studies [6,10]. Female partners enrolled in a large survey of 121 infertile couples reported low mean scores in FSFI, 26% of which were consistent with high risk of sexual dysfunction [10]. Furthermore Laffont’s study showed a marked drop in sexual desire in the women enrolled [33]. Finally, according to the results of Ohl’s research, women experience less sexual satisfaction compared to their partners and avoid sexual intercourse more frequently [7].

Focusing on the difference between the groups, we might speculate a different impact of the stage of treatment. As far as IIEF is concerned, Group A scored lower than the controls in the “erectile dysfunction” subscale. This datum highlights the importance of an infertility diagnosis on male sexual function. According to the literature, temporary erectile disorders could often be caused by the diagnostic exams, such as semen analyses and post-coital tests. A meaningful finding in this respect is that in one study 11% of men with a previously abnormal semen analysis are unable to produce the sperm needed for a second spermiogram [34]. Another significant difference between Group A and normal controls were the decline in “orgasm”, “desire”, “sexual satisfaction” and “overall satisfaction” in the former. These can be due to the progressive erotic disinvestment of sexual activity, enhanced by medical prescriptions that encourage patients to have sex preferably on fertile days [8]. As far as FSFI is concerned, most of the parameters analyzed were significantly lower in infertile patients if compared to fertile controls: sexual intercourse is deprived of its erotic value, thus it is characterized by fewer preliminaries, lower sexual desire, poorer perception of psychic excitation and fewer signs of physical excitation.

The current study findings should be interpreted considering some methodological limitations. First of all, the small sample size could have prevented us from finding statistically significant differences in some of the fields explored. Moreover, an adequate follow-up was not carried out, thus possible consequences of infertility treatment might have been underestimated. Further studies on bigger samples and longer follow-up will settle the issue. Furthermore objections could be raised to the instruments used in the present study. Since no study has proved the relationship between FSFI/IIEF and personality measures yet, still the latter are not universally recognized as predictors of sexual functioning. On the other hand, psychopathological symptoms related to infertility, like anxiety, hostility, depression have not been directly evaluated in the present study, although they are known to affect sexual functioning. Thus the differences found between infertile couples and non-infertile couples cannot be completely attributed to infertility or to the psychological maladjustment resulting from infertility. Additionally it can be argued that, since our control group was not looking for pregnancy (right now/ in the short term), the erotic valence of sex would be potentially higher in this group (that has more freedom to decide when and how to have sex), possibly affecting the scores on FSFI/IIEF (particularly sexual desire, lubrication, female orgasm). Besides no distinctions were made on the grounds of the duration of infertility; it would be useful, instead, to examine possible differences depending on the duration of involuntary childlessness. What is more, our results can be regarded as partial because the couples enrolled were candidate to IUI. Thus it can be argued that our sample was made up of patients suffering from milder infertility conditions that do not require complex techniques, so they might have had a more optimistic view of their situation and have experienced lower sexual and emotional distress. On the other hand most of the studies on this topic engage patients undergoing Intracytoplasmic sperm injection (ICSI) or in vitro fertilization and embryo transfer (IVF/ET). Besides, our data proved to be useful in assessing two important dimensions of the infertile couple’s background: sexuality and personality. Furthermore they provide a useful “portrait” of the infertile couple in the very first phase of the treatment. Even if the sexual disturbances detected differed both according to the gender and to the phase of treatment, they overall showed a strong relation to infertility and its treatment. Thus sexual counseling should be introduced in the clinical management of infertile couples in order to preserve the quality of the couple’s sexual relationship and optimize the chances of pregnancy. In fact many studies have claimed the importance of a global approach to the childless couples, in which the systematical discussion of sexual problems should be granted [4,7,10]. Furthermore sexual counseling could help prevent psycho-sexual repercussions, favoring the couple’s adjustment to infertility diagnosis and medical treatment, and it could point out possible distress caused by sexual disturbances. Above all, sexual counseling should aim at restoring the playful, spontaneous, and imaginative side of sexuality, regardless of the fertile days [4].

## Conclusion

Our study has pointed out the relevance of sexuality and personality features in the clinical management of infertile couples. Even if no distinguishing personality feature could be pointed out, it is clear that infertile patients experience great stress even in the very first phase of treatment with a heavy impact on their sexual activity, especially on sexual desire and sexual arousal, with women more affected than men. Thus physicians should take into due consideration the possible sexual difficulties of couples to avoid a vicious cycle that reduces the likelihood of pregnancy and could potentially lead to a permanent disruption of the sexual relationship.

## Abbreviations

IUI, Intrauterine Insemination; DSM-IV, Diagnostic and statistical manual of mental health disorders, 4th edition; ACL, Adjective Check List; IIEF, International Index of Erectile Function; FSFI, Female Sexual Function Index.

## Competing interests

Authors declare to have no commercial and/or financial interest with manufacturers of pharmaceuticals, laboratory supplies, and/or medical devices; they declare to have no relationship with commercial providers of medically related services.

## Authors’ contribution

RM has substantially contributed to data collection, to the diagnostic process, to the preoperative and operative work up linked to the condition of the patient, to preparation, drafting and revising the final version of the manuscript. He also gave an extremely important intellectual support. AG has substantially contributed to design, to preparation, drafting and revising the final version of the manuscript and gave and significant intellectual support. IP has substantially contributed to design, to preparation, drafting and revising the final version of the manuscript and gave and significant intellectual support.

Lo Monte G has substantially contributed to design, preparation, drafting and revising the intellectual content of the final version of the manuscript. IS has substantially contributed to design, preparation, drafting and revising the intellectual content of the final version of the manuscript. EG has substantially contributed to design, preparation, drafting and revising the intellectual content of the final version of the manuscript. SM has substantially contributed to design, to preparation, drafting and revising the final version of the manuscript and gave and significant intellectual support. CR has substantially contributed to design, preparation, drafting and revising the intellectual content of the final version of the manuscript. MC has substantially contributed to data collection, to the diagnostic process, to the preoperative and operative work up linked to the condition of the patient, to preparation, drafting and revising the final version of the manuscript. He also gave an extremely important intellectual support. SC has substantially contributed to data collection, to the diagnostic process, to the preoperative and operative work up linked to the condition of the patient, to preparation, drafting and revising the final version of the manuscript. He also gave an extremely important intellectual support. AP has substantially contributed to data collection, to the diagnostic process, to the preoperative and operative work up linked to the condition of the patient, to preparation, drafting and revising the final version of the manuscript. He also gave an extremely important intellectual support. All authors read and approved the final manuscript.

## References

[B1] Zegers-Hochschild F Adamson GD de Mouzon J Ishihara O Mansour R Nygren K Sullivan E Vanderpoel S International Committee for Monitoring Assisted Reproductive Technology; World Health Organization. International Committee for Monitoring Assisted Reproductive Technology (ICMART) and the World Health Organization (WHO) revised glossary of ART terminology Fertil Steril 2009 92 1520 1524 10.1016/j.fertnstert.2009.09.009 19828144

[B2] Wischmann TH Psychogenic infertility–myths and facts J Assist Reprod Genet 2003 20 485 494 15035547 10.1023/B:JARG.0000013648.74404.9d PMC3455307

[B3] Nene UA Coyaji K Apte H Infertility: a label of choice in the case of sexually dysfunctional couples Patient Educ Couns 2005 59 234 238 10.1016/j.pec.2005.08.005 16242294

[B4] Wischmann TH Sexual disorders in infertile couples J Sex Med 2010 7 1868 1876 10.1111/j.1743-6109.2010.01717.x 20214712

[B5] Cousineau TM Domar AD Psychological impact of infertility Best Pract Res Clin Obstet Gynaecol 2007 21 293 308 10.1016/j.bpobgyn.2006.12.003 17241818

[B6] Wirtberg I Möller A Hogström L Tronstad SE Lalos A Life 20 years after unsuccessful infertility treatment Hum Reprod 2007 22 598 604 17124258 10.1093/humrep/del401

[B7] Ohl J Reder F Fernandez A Bettahar-Lebugle K Rongières C Nisand I Impact of infertility and assisted reproductive techniques on sexuality Gynecol Obstet Fertil 2009 37 25 32 10.1016/j.gyobfe.2008.08.012 19117786

[B8] Coëffin-Driol C Giami A L’impact de l’infertilité et de ses traitments sur la vie sexuelle et la relation de couple: revue de la literature Gynecol Obstet Fertil 2004 32 624 637 10.1016/j.gyobfe.2004.06.004 15450262

[B9] Greil AL Infertility and psychological distress: a critical review of the literature Soc Sci Med 1997 45 1679 1704 10.1016/S0277-9536(97)00102-0 9428088

[B10] Nelson CJ Shindel AW Naughton CK Ohebshalom M Mulhall JP Prevalence and predictors of sexual problems, relationship stress, and depression in female partners of infertile couples J Sex Med 2008 5 1907 1914 10.1111/j.1743-6109.2008.00880.x 18564149

[B11] Seibel M Taymore M Emotional aspects of infertility Fertil Steril 1982 37 137 145 7037462 10.1016/s0015-0282(16)46029-2

[B12] Cloninger CR Svrakic DM Przybeck TR A psychobiological model of temperament and character Arch Gen Psychiatry 1993 50 975 990 10.1001/archpsyc.1993.01820240059008 8250684

[B13] Fassino S Garzaro L Peris C Amianto F Pierò A Abbate Daga G Temperament and character in couplet with fertility disorders: a double-blind, controlled study Fertil Steril 2002 77 1233 1240 10.1016/S0015-0282(02)03115-1 12057734

[B14] Rothman D Kaplan DH Nettles E Psychosomatic infertility Am J Obstet Gynecol 1962 83 373 378 14494437 10.1016/s0002-9378(16)35844-6

[B15] Csemiczky G Landgren BM Collins A The influence of stress and state anxiety on the outcome of IVF-treatment: psychological and endocrinological assessment of Swedish women entering IVF-treatment Acta Obstet Gynecol Scand 2000 79 113 118 10.1034/j.1600-0412.2000.079002113.x 10696958

[B16] American Psychiatric Association Diagnostic and statistical manual of mental health disorders (4th ed) 1994 Washington DC: American Psychiatric Association

[B17] Epstein YM Rosenberg HS Depression in primary versus secondary infertility egg recipients Fertil Steril 2005 83 1882 1884 10.1016/j.fertnstert.2005.01.098 15950673

[B18] Lerman H Questions of validity raised by an adjective check list technique J Clin Psychol 1963 19 198 199 10.1002/1097-4679(196304)19:2<198::AID-JCLP2270190215>3.0.CO;2-G 13929832

[B19] Zuckerman M Lubin B Normative data for the multiple affect adjective check list Psychol Rep 1965 16 438 10.2466/pr0.1965.16.2.438 14285844

[B20] Bloom PM Brady JP An ipsative validation of the multiple affect adjective check list J Clin Psychol 1968 24 45 46 10.1002/1097-4679(196801)24:1<45::AID-JCLP2270240112>3.0.CO;2-0 5639458

[B21] Gough HG Heilbrun AB Fioravanti M L’Adjective Check list 1980 Firenze: Organizzazioni Speciali

[B22] Herron EW The multiple affect adjective check list: a critical analysis J Clin Psychol 1969 25 46 53 10.1002/1097-4679(196901)25:1<46::AID-JCLP2270250113>3.0.CO;2-O 5782333

[B23] Itzhar-Nabarro Z Silberschatz G Curtis JT The adjective check list as an outcome measure: assessment of personality change in psychotherapy Psychother Res 2009 19 6 707 717 10.1080/10503300902988760 19606389

[B24] Rosen RC Riley A Wagner G Osterloh IH Kirkpatrick J Mishra A The international index of erectile function (IIEF): a multidimensional scale for assessment of erectile dysfunction Urology 1997 49 822 830 10.1016/S0090-4295(97)00238-0 9187685

[B25] Rosen RC Cappelleri JC Gendrano N The international index of erectile function (IIEF): a state-of-the-science review Int J Impot Res 2002 14 226 244 10.1038/sj.ijir.3900857 12152111

[B26] Rosen R Brown C Heiman J Leiblum S Meston C Shabsigh R Ferguson D D'Agostino R The female sexual function index (FSFI): a multidimensional self-reported instrument for the assessment of female sexual function J Sex Marital Ther 2000 26 191 208 10.1080/009262300278597 10782451

[B27] Nappi RE Albani F Vaccaro P Gardella B Salonia A Chiovato L Spinillo A Polatti F Use of the Italian translation of the Female Sexual Function Index (FSFI) in routine gynecological practice Gynecol Endocrinol 2008 24 214 219 10.1080/09513590801925596 18382908

[B28] Monga M Alexandrescu B Katz SE Stein M Ganiats T Impact of infertility on quality of life, marital adjustment, and sexual function Urology 2004 63 126 130 10.1016/j.urology.2003.09.015 14751363

[B29] Anderson KM Sharpe M Rattray A Irvine DS Distress and concerns in couples referred to a specialist infertility clinic J Psychosom Res 2003 54 353 355 10.1016/S0022-3999(02)00398-7 12670613

[B30] Hamberger L Janson PO Global importance of infertility and its treatment: role of fertility technologies Int J Gynaecol Obstet 1997 58 149 158 10.1016/S0020-7292(97)00286-5 9253677

[B31] Wright J Duchesne C Sabourin S Bissonnette F Benoit J Girard Y Psychosocial distress and infertility: men and women respond differently Fertil Steril 1991 55 100 108 1986949

[B32] Benazon N Wright J Sabourin S Stress, sexual satisfaction, and marital adjustment in infertile couples J Sex Marital Ther 1992 18 273 284 10.1080/00926239208412852 1291698

[B33] Laffont I Edelmann RJ Psychological aspects of in vitro fertilization: a gender comparison J Psychosom Obstet Gynaecol 1994 15 85 92 10.3109/01674829409025633 7921010

[B34] Saleh RA Ranga GM Raina R Nelson DR Agarwal A Sexual dysfunction in men undergoing infertility evaluation: a cohort observational study Fertil Steril 2003 79 909 912 10.1016/S0015-0282(02)04921-X 12749429

